# Trends and risk factors of stillbirth among women of reproductive age in Pakistan: A multivariate decomposition analysis

**DOI:** 10.3389/fpubh.2023.1050136

**Published:** 2023-02-23

**Authors:** Abeera Shakeel, Asifa Kamal, Muhammad Ijaz, Maryam Siddiqa, Getayeneh Antehunegn Tesema, Tahani Abushal

**Affiliations:** ^1^Department of Statistics, Lahore College for Women University, Lahore, Pakistan; ^2^Department of Mathematics and Statistics, The University of Haripur, Haripur, Pakistan; ^3^Department of Mathematics and Statistics, International Islamic University Islamabad, Islamabad, Pakistan; ^4^Department of Epidemiology and Biostatistics, Institute of Public Health, College of Medicine and Health Sciences, Comprehensive Specialized Hospital, University of Gondar, Gondar, Ethiopia; ^5^Department of Mathematical Sciences, Umm Al-Qura University, Makkah al Mukarramah, Saudi Arabia

**Keywords:** stillbirth, MVDCMP, multivariate decomposition, ENAP, Pakistan

## Abstract

**Background:**

Every year, 2 million babies are stillborn in the world. Globally, there has been a decline in the stillbirth rate of 2%. Despite advancements in prenatal care and the implementation of new medical technologies, the incidence of early stillbirths remains unchanged. A slight decrease in the rate of late-term stillbirth has been observed. Pakistan ranked third in South Asia for having the highest stillbirth rate. Compared to its neighbors and other developing nations, Pakistan has shown a lack of progress in reducing maternal and neonatal fatalities. Therefore, the purpose of this study is to use a multivariate decomposition analysis to examine the trends and factors that have contributed to the change in the stillbirth rate over time.

**Methods:**

To conduct this study, we used a secondary data analysis approach and analyzed data from the Pakistan Demographic and Health Survey (PDHS) of 2012–2013 and 2017–2018). For the analysis, a total sample of 15,068 births in 2017–2018 and 13,558 births in the PDHS from 2012 to 2013 were taken into account. Using the MVDCMP function within STATA version 15 statistical software, a logit-based multivariate decomposition model was fitted to determine the variables that influence the change in stillbirth. The current study used two cross-sectional surveys to identify important risk factors for stillbirths.

**Results:**

Over the past 5 years, Pakistan's stillbirth rate has risen from 3.98 to 5.75%. According to the total multivariate decomposition analysis, the change in coefficient (change in the effect of attributes) accounted for 81.17% of the overall change in the proportion of stillbirths. In contrast, the change in endowment was not statistically significant. Changes in maternal education, individual and community-level wealth status, and mode of delivery all significantly impacted the rate of stillbirths over time.

**Conclusion:**

Stillbirths increased in Pakistan from 2012 to 2017. Stillbirths are observed more frequently for women residing in Punjab, Sindh, and rural areas. A major concern that is directly related to the prevalence of stillbirths in Pakistan is the lack of accessible, affordable, and high-quality maternal healthcare facilities. Older, overweight, and uneducated women are more likely to have stillbirths than women who deliver vaginally. High parity and short birth intervals also accelerated the rate of stillbirths. An effective remedy to control stillbirths is the provision of accessible and affordable healthcare services. Awareness campaigns for the health education of pregnant women should focus on raising awareness to support better pregnancy outcomes for poor women living in communities with higher education levels. The risk of stillbirth can be reduced by offering free diagnostics for early detection of birth complications in low-resource settings and referring these cases to knowledgeable gynecologists for safe delivery.

## Introduction

Approximately 2 million babies are stillborn worldwide annually (1). The highest incidence of stillbirths, with more than 30 stillbirths per 1,000 live births, is observed in Southeast Asia and sub-Saharan Africa (SSA) (2, 3). Globally, the stillbirth rate decreased by 2.0% (4). The past two decades have seen a 35% decrease, globally in stillbirths (5). Even though prenatal care services have improved and new medical technology has been introduced, early stillbirths have not decreased. However, the rate of late-term stillbirths has decreased slightly (6). Globally, 2.6 million stillbirths are estimated every year. The risk of stillbirths is 20 times higher for women in low- and middle-income countries than for women in high-income countries. The majority of Asian and sub-Saharan countries bear the burden of stillbirths. A study also reported, Pakistan as one of the highest stillbirth rates among South Asian countries, with a rate of 30.6 per 1,000 total births, which is significantly higher than the average for South Asia, which is 18.2 per 1,000 total births (7). Similarly, the stillbirth rate in India is also lower, at 13.9 per 1,000 total births (8).

There are different definitions of in different nation stillbirth in different countries (9). Pakistan adheres to the definition recommended by the World Health Organization (WHO), which is for international comparison. A stillbirth occurs when a fetus dies after seven months of gestation (10).

Pakistan is the third country in South Asia with the highest stillbirth rate (11). Pakistan had 43.1 stillbirths per 1,000 live births in 2015, compared to an estimated 18.4 stillbirths per 1,000 live births globally (12, 13). Compared to its neighbors and other nations, Pakistan developing countries, Pakistan, Pakistan had limited success in lowering maternal and neonatal fatalities (14). The UN and WHO announced the ENAP strategy, which aims to reduce stillbirths to < 12 per 1,000 births by 2019 (15). Stillbirth is still a significant problem in Pakistan. Despite the high prevalence of stillbirths in Pakistan, the issue remains unrecognized by healthcare policymakers. Underreporting these issues due to social taboos is one of the reasons for this unrecognized health problem (13). Pakistani women are reluctant to discuss stillbirths for fear of being stigmatized as infertile or being subject to misconceptions such as the influence of an evil eye or black magic (16). Globally, stillbirths were not a priority within the Millennium Development Goals (MDGs). However, the Every New Born Action Plan (ENAP) highlighted the significance of the issue. Poor maternal health, insufficient gynecological and obstetric care, and insufficient delivery methods may all contribute to high stillbirth rates (17).

Pregnancy complications are the main cause of stillbirth. Intrauterine infection, birth asphyxia, hypertension, eclampsia, preterm labor, and insufficient prenatal care are significant contributors to stillbirth (18). The most common causes of stillbirth include bleeding before or during labor, placental abruption, infections, birth defects, poor lifestyle choices, lupus, clotting disorders, trauma, intrahepatic cholestasis of pregnancy (ICP), or obstetric cholestasis (19). In Pakistan, hypertension has been observed to be the main cause of stillbirths (20).

Many causes of stillbirth have not been identified. Therefore, it is the job of healthcare professionals to devise strategies to mitigate its risks (21). Early infection detection can also reduce stillbirth rates (22). Many sociodemographic factors significantly influenced the rise in stillbirth rates (23). Residence, wealth index, maternal age, education, employment status, birth order, birth interval, antenatal care visit, place of delivery, and mode of delivery are among the factors that have been identified as significant potential causes of stillbirth (24–27). Tracking the fluctuations in stillbirth rates over time is difficult because current studies only analyze one specific point. Using data from the national survey, the Pakistan Demographic and Health Survey (PDHS), this study sought to provide a detailed understanding of the factors that lead to stillbirth in Pakistan (PDHS 2012–2013 and 2017–2018). As reducing stillbirth is somewhat related to decreasing maternal and neonatal deaths, this research is crucial (28). A multivariate decomposition technique that identifies the determinants connected with the change over time was utilized to analyze the change in stillbirth. Health ministries can benefit from this study's recommendations for better interventions to address the reasons for Pakistan's rising stillbirth rate. Therefore, this study aimed to investigate the magnitude, trends, and factors that contributed to the change in stillbirth rate over time using a multivariate decomposition.

## Materials and methods

### Data

This study is based on secondary data analysis of the PDHS for the years 2012 to 2013 and from 2017 to 2018.

### Sample and population

The PDHS used a two-stage stratified clustered sampling design. A structured sampling approach was employed to select households randomly. The questionnaire was completed by 13,558 of the 14,569 ever-married women who were chosen as the sample in the PDHS from 2012 to 2013. The PDHS from 2017 to 2018 is Pakistan's fourth demographic and health survey. A sample of 15,671 eligible, ever-married women between the ages of 15 and 49 years was chosen using the same selection technique as the PDHS from 2012 to 2013. In the PDHS from 2017 to 2018, 15,068 women responded.

### Study variables

#### Outcome variable

The stillbirth rate among mothers of reproductive age served as the study's primary outcome variable. Stillbirth is defined as the death of a fetus during the third trimester of pregnancy (≥28 weeks) (10). The response variable for the one woman who had a stillbirth was entered as 1, while the response variable for the other mothers was coded as 0.

#### Explanatory variable

In the current study, ever-married women's sociodemographic traits, neighborhood-level variables, and labor and delivery-related traits were treated as independent variables. Maternal age, education, employment status, and the father's education, wealth index, and media exposure were considered sociodemographic factors. Maternal Body Mass Index (BMI), maternal height, birth order, prior birth interval, number of ANC visits, delivery venue, delivery style, place of delivery, and mode of delivery were considered labor and delivery care factors.

This study employed a data aggregation method at the individual level of each primary sampling unit (PSU) to compute community-level variables. This study examined community-level factors, including maternal education, financial status, and media exposure. These factors were divided into high- and low-level groups in relation to the national mean value. The national mean value of each variable, which was divided into high and low levels, was used to aggregate community-level variables. At the cluster level, the education level, income, and media exposure of the mother were added and compared to their averages.

### Data collection procedure

The official DHS measure website was used to retrieve the PDHS 2017-2018 and PDHS 2012-2013 statistics (29, 30). The outcome and independent factors were collected from the birth record file used in this investigation.

### Statistical analysis

The study aimed to evaluate the individual contributions of various factors through the use of descriptive and multivariate analysis.

### Descriptive analysis

The descriptive analysis included the calculation of the percentage distribution of the selected characteristics of ever-married women in both surveys and the proportion of respondents who experienced stillbirth, showing a change in the prevalence of stillbirth from 2012 to 2017.

### Multivariate decomposition model

Oaxaca–Blinder introduced the concept of decomposition of linear regression in econometrics for the first time. It is also called regression standardization. The idea of regression decomposition was also expanded to include nonlinear regression models. A multivariate decomposition technique was used to quantify the change in stillbirth rates and identify the key factors that contributed to the rise in stillbirth over the study period. Numerous scholars employed multivariate decomposition analysis for linear regression models in the 1970s and later extended it to include nonlinear regression models (31). For the nonlinear response model, logistic-based decomposition analysis was selected because the outcome variable was binary. Logit models were developed by Fairlie (32), Nielsen (33), Bowblis, and Yun (34). This technique provides a method to analyze the outcomes of two separate groups. The logistic decomposition model was used to determine the factors contributing to the change in stillbirth over the last 5 years. In decomposition models, the difference between two groups is explained by the difference in the mean, proportion, or counts. That difference in the mean, proportion, or count is decomposed into two parts. One is called composition, endowments, or characteristics, and the other is called a coefficient or the effects of those components. In this approach, the actual contribution of each predictor to the total difference is due to the composition of characteristics or the effects of characteristics.

The difference in endowments between surveys and the influence of different explanatory variables can be considered responsible for the shift in stillbirth over time. The observed variation in stillbirth between surveys is broken down into components for the composition of characteristics and the coefficients of characteristics.

The decomposition model output provides detail on endowments and coefficients between two time periods:

- Endowment: The change in stillbirth that is due to the difference in characteristics.
- Coefficients: The change in stillbirth that is because of explanatory variables.

The decomposition model for the current study is expressed as follows:


(1) $$\begin{array}{lll} {\bar{Y}}_{2017}-{\bar{Y}}_{2012} & = & \bar{F\left(X_{2017}\beta_{2017}\right)}-\bar{F\left(X_{2012}\beta_{2012}\right)} \end{array}$$

$$\begin{array}{lll} {\bar{Y}}_{2017}-{\bar{Y}}_{2012} & = & \bar{F\left(X_{2017}\beta_{2017}\right)}-\bar{F\left(X_{2012}\beta_{2017}\right)}+\bar{F\left(X_{2012}\beta_{2017}\right)} \end{array}$$

(2) $$\begin{array}{ll} - & \bar{F\left(X_{2012}\beta_{2012}\right)} \end{array}$$

$$\begin{array}{lll} {\bar{Y}}_{2017}-{\bar{Y}}_{2012} & = & \left({\bar{X}}_{2017}-{\bar{X}}_{2012}\right)\beta_{2017}+\left(\beta_{2017}-\beta_{2012}\right){\bar{X}}_{2012}\\ = & Differences\;in\;Endowement\;part \end{array}$$

(3) $$\begin{array}{ll} + & Differences\;in\;Coefficients\;part \end{array}$$

The first part of differential attributable (3) is the explained component or characteristic component, so this difference is called endowments or characteristics. It shows the change in the prevalence of a set of predictors/indicators at both time points (35). The second part of the differential attributable (3) is a generally unexplained component and refers to the difference in coefficients or effects. It is a change in the effect of these predictors/indicators measured as a coefficient effect at both time points (35, 36).

Where, ${\bar{Y}}_{2017}-{\bar{Y}}_{2012}$ is the difference in mean prediction between 2017 and 2012, *X*_*i*_, …….., *X*_*k*_ are the difference in characteristics, and β_*i*_, ………, β_*k*_ are the estimated regression coefficients. However, $\left({\bar{X}}_{2017}-{\bar{X}}_{2012}\right)\beta_{2017}$ represents differences due to characteristics or endowments and $\left(\beta_{2017}-\beta_{2012}\right){\bar{X}}_{2012}$ represents differences due to coefficients.

To apply the above model, the MVDCMP STATA command was used, which provides the effect of each covariate (36).

The current study employed the multivariate decomposition approach for two main reasons. The first objective is to compare stillbirth rates between the two time periods of 2012 and 2017. The second reason is that the multivariate decomposition model allows for an examination of the difference in stillbirths between 2012 and 2017, broken down into two components: changes in the composition of the population and changes in the effects of independent variables.

The application of the multivariate decomposition method requires two data sets with the same factors or indicators at two points in time (31). The PDHS data sets provide data for the same indicator in two consecutive waves, i.e., PDHS 2017–2018 and PDHS 2012–2013. In the literature, data sets collected at different points in time from the same country using the Demographic and Health Survey (DHS) have been utilized to apply a multivariate decomposition regression model (35).

### Ethical considerations

The PDHS is regarded as a nationally representative survey. The sample size and power of the study were good in the PDHS from 2012 to 2013 and PDHS from 2017 to 2018. DHS data are publicly available for free, and no ethical approval is required. Access to the data was gained on the Demographic and Health Survey website *via* an online request. The data were released following the approval of the DHS measures letter.

## Results

### Sociodemographic characteristics of ever-married women

Women who had children in the 5 years before the survey were included in the current study. Sociodemographic variable percentage distributions for the PDHS from 2017 to 2018 and the PDHS from 2012 to 2013 are given in Table 1. In both surveys, almost two-thirds more births were reported in rural areas than in urban areas.

**Table 1:** Sociodemographic characteristics of ever married women who had given birth within 5 years before survey in Pakistan, PDHS from 2012 to 2013 and from 2017 to 2018.

**Variables**	**PDHS (2012–2013)**		**PDHS (2017–2018)**	
	***N***	**%**	***N***	**%**
**Place of Residence**				
Urban	3,416	28.4	3,348	32.7
Rural	8,613	71.6	6,879	67.3
**Region**				
Punjab	6,899	57.8	5,395	52.8
Sindh	2,700	22.6	2,370	23.2
KPK	1,690	14.2	1,605	17.9
Balochistan	605	5.07	553	5.40
ICT	49	0.41	75	0.74
**Wealth Index**				
Poor	5,550	46.1	4,407	43.1
Middle	2,334	19.4	2,134	20.9
Rich	4,146	34.5	3,685	36
**Maternal Age**				
15-24	2,093	17.4	1,787	17.5
25-29	3,721	30.9	3,266	31.9
30+	6,215	51.7	5,173	50.6
**Maternal Education**				
No Education	7,113	59.1	5,095	49.82
Primary	1,978	16.4	1,718	16.8
Secondary	2,008	16.7	2,183	21.4
Higher	930	7.73	1,230	12.03
**Maternal Work Status**				
Unemployed	8,652	72.1	8,560	83.7
Employed	3,341	27.9	16,656	16.3
**Husband Education**				
Un educated	6,220	51.9	4,747	47.3
Educated	5,769	48.1	5,288	52.7

Regarding maternal age, most women (51%) who had given birth were found to be at least 30 years old. In both surveys, most women (59.1 and 49.8%) who had given birth within the previous 5 years were uneducated. In the later survey, the percentage of educated women had slightly increased compared to the earlier one. The proportion of unemployed women who had given birth was greater (84%) in the PDHS from 2017 to 2018 than it was in the preceding survey (72%) (2012–2013). The percentage of employed mothers was lower in the recent survey than in the preceding survey.

### Labor, delivery care and other characteristics of ever-married women

Table 2 clearly shows that according to the PDHS from 2012 to 2013, out of 12,029 women, only 67% of women had media exposure. A slight decrease in the percentage (60.3%) was observed for the PDHS from 2017 to 2018, with a total of 10,226 women. The percentage of overweight women was found to be higher than underweight in both surveys. Births were higher in a recent survey (46%), compared to an older survey (32.8%), for overweight women. Over time, an increase of 13.2 was observed in the number of births among obese mothers. The majority of newborns had mothers who were 150 cm or taller.

**Table 2:** Labor, Delivery Care and Other Characteristics of Ever Married Women who had given birth within 5 years before survey in Pakistan, PDHS from 2012 to 2013 and from 2017 to 2018.

**Variables**	**PDHS (2012–2013)**		**PDHS (2017–2018)**	
	***N***	**%**	***N***	**%**
**Media Exposure**				
No	3,988	33.3	4,057	39.7
Yes	8,028	66.7	6,169	60.3
**Maternal BMI**				
Underweight	617	14.5	382	10.1
Normal	2,240	52.7	1,661	43.9
Overweight	1,397	32.8	1,742	46.0
**Women's Height**				
<150 cm	833	19.5	788	20.8
≧150 cm	3,433	80.5	3,000	79.2
**Birth Order**				
1-3	7,015	58.3	6,411	62.7
4-6	3,494	29.0	2,930	28.7
7+	1,520	12.6	884	8.64
**Preceding Birth Interval**				
<24 Months	3,530	38.1	3,011	39.0
24-36 Months	3,161	34.15	2,508	32.5
48+ Months	2,566	27.72	2,207	28.6
**ANC Visit**				
No Visit	1,337	11.1	572	5.59
1-3	1,963	16.3	1,642	16.1
4+	8,729	72.6	8,012	78.4
**Place of Delivery**				
Home	5,164	53.5	2,913	35.1
Health Facility	4,484	46.5	5,383	64.9
**Mode of Delivery**				
Vaginal	8,384	86.9	6,529	83.1
Cesarean section	1,265	13.1	1,766	16.9
**Community Maternal Education**				
Low Level	8,163	67.9	5,864	57.3
High Level	3,866	32.1	4,361	42.7
**Community Wealth Index**				
Low Level	6,259	52.0	4,784	46.8
High Level	5,770	48.0	5,442	53.2
**Community Media Exposure**				
Low Level	6,024	50.1	4,611	47.9
High Level	6,006	49.9	5,614	52.2

According to a recent survey, the percentage of children with birth orders older than seven has decreased. A significant reduction (−3.96) was seen for mothers with more than seven children. In the most recent study (78.4%), compared to the previous survey (72.6%), there was a modest increase in the percentage of children born to women who had received antenatal care more than four times. Compared to mothers who had given birth at home, there was an increase of 18.4% over time for mothers who had given birth in a medical institution. Almost four-fifth (83.1%) of Pakistani women gave birth naturally, whereas only one in every sixteen respondents (16.9%) had a cesarean section in the PDHS (2017–2018).

In both surveys, the majority of women (67.9 and 57.3%) who had given birth in the previous 5 years were found to live in localities with lower levels of education. Women who resided in neighborhoods with lower levels of wealth showed a 46.8% in the most recent PDHS survey compared to the previous survey (52.0%). In the prior survey, nearly 50% of the women interviewed lived in areas with less media exposure. The percentage in the most recent survey marginally decreased for this low category of media exposure (45.1%). exposure (47.9%).

### The trend in stillbirth from the PDHS 2012–2013 and PDHS 2017–2018 in Pakistan

**Figure 1:**
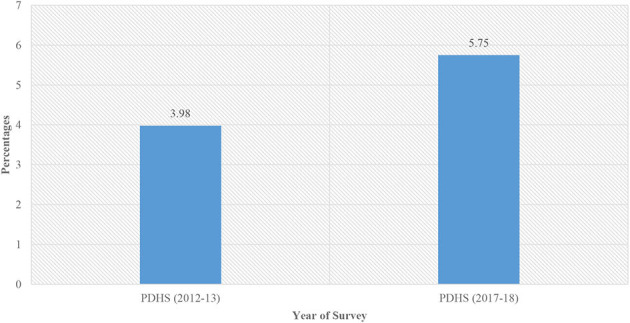
Trend in stillbirth in Pakistan from the PDHS from 2012 to 2013 to the PDHS from 2017 to 2018.

Figure 1 shows that stillbirths increased in Pakistan from the PDHS from 2012 to 2013 (3.98%) to the PDHS from 2017 to 2018 (5.75%).

### Trends in stillbirth percentage by selected sociodemographic characteristics in Pakistan

**Figure 2:**
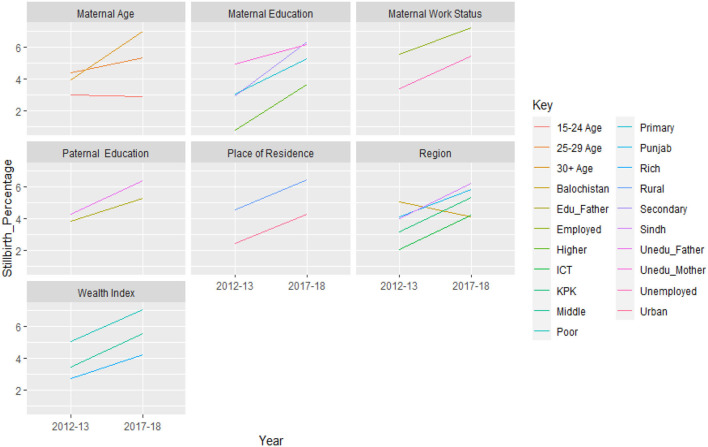
Trend in the stillbirth rate between surveys, by selected sociodemographic characteristics, (PDHS 2012–2013 and PDHS 2017–2018).

Figure 2 displays trends in stillbirth by respondents' sociodemographic details from two PDHS surveys (2012–2013 and 2017–2018). Each group of sociodemographic characteristics revealed variation in the trend in the prevalence of stillbirths. Punjab and Sindh regions, rural areas, mothers with low socioeconomic and education levels, and mothers who were 25 or older all showed an increase in the percentage of stillbirths from the PDHS from 2012 to 2013 to the PDHS from 2017 to 2018. The percentage of stillbirths increased in rural areas according to the location of residence, showing that stillbirths were more likely to occur in Pakistan's rural areas. Stillbirth rates were higher in Punjab and Sindh than in other regions. Women from low- and middle-income backgrounds had a significant rise in stillbirths. Stillbirth rates had increased significantly among mothers with lower levels of education. However, from the PDHS from 2012 to 2013 to the PDHS from 2017 to 2018, mothers with higher education demonstrated a low prevalence of stillbirth. Over the course of 5 years, the stillbirth rate among working mothers increased. The percentage of stillbirths among younger women (under 30 of age) increased during two surveys. While the risk of stillbirth among older women was low in the PDHS from 2012 to 2013, it showed a rise in the PDHS from 2017 to 2018. The stillbirth rate increased over time for children whose fathers had lower levels of education, but for children whose fathers had higher levels of education, the stillbirth rate decreased.

### The trend in stillbirth percentage by selected Labor, delivery care and other characteristics Pakistan

The percentage of stillbirths increased for overweight mothers compared to underweight or normal mothers. Compared to underweight and normal mothers, those who were overweight experienced higher incidences of stillbirths. When both surveys were considered, stillbirths were more common among mothers shorter than 150 cm or those taller than 150 cm. A significant rise in the stillbirth rate in the PDHS from 2017 to 2018 was seen among women who had become pregnant after six births as compared to the PDHS from 2012 to 2013. Compared to women who gave birth before 24 months than those who became pregnant after that time were more likely to have stillbirths.

Both studies showed a high percentage of stillbirths among women who did not receive any prenatal care. A surprising finding regarding the place of delivery was that stillbirth rates were higher among women who delivered in a hospital compared to those who delivered at home; however, the stillbirth rate was lower for women who delivered *via* cesarean section compared to those who gave birth vaginally.

Women's education at the individual level was divided into two categories: low (no education or primary education) and high (secondary or higher education). Similar to how low levels of the wealth index were classified as “poorer or worst wealth status” and higher levels as “middle, higher, and highest wealth status.” Individual women who had no media exposure were regarded as having a low level of media exposure, whereas those who had media exposure were regarded as having a high degree of media exposure. Compared to women who lived in communities with high levels of education, those who lived in low-educated communities reported having higher incidences of stillbirths.

**Figure 3:**
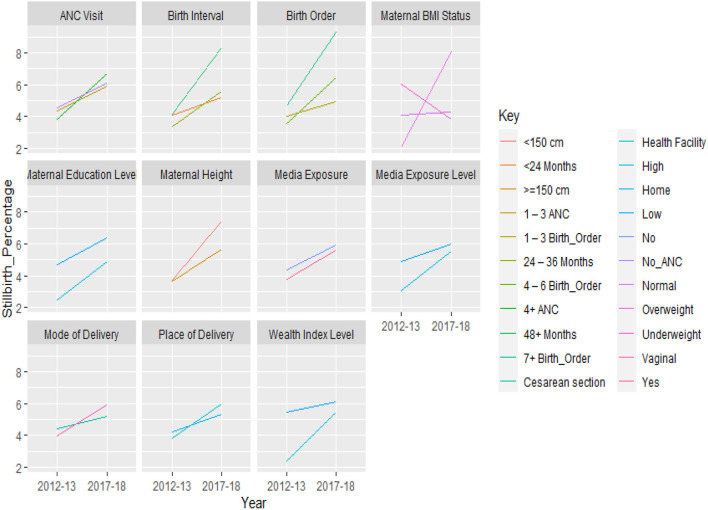
Trend in the stillbirth rate between surveys, by selected labor, delivery care and other characteristics, (PDHS 2012–2013 and PDHS 2017–2018).

Figure 3 shows that women in low-wealth index communities experienced stillbirths more frequently in the PDHS from 2012 to 2013 than women in high-wealth index communities. Compared to individuals who were significantly exposed to media, communities with low levels of media exposure were found to have higher percentages of stillbirths.

### Decomposition analysis

Changes in the prevalence of stillbirths were divided into two parts. The first part identified the change in composition or characteristics of the selected variables. In comparison, the second part described the change due to the coefficients of those variables. Table 3 shows the overall decomposition analysis of the decrease or increase in stillbirth over the last 5 years.

**Table 3:** Overall multivariate decomposition analysis result of the decrease in stillbirths, PDHS from 2012 to 2013 and from 2017 and 2018.

**Stillbirths**	**Coefficient**	**Percentage (%)**	***P*** **-value**	**95% confidence limits**	
				**Lower**	**Upper**
Endowments	0.006204	18.83	0.103	−0.0012	0.0137
Coefficients	0.026742	81.17	0.000	0.0130	0.0405
R	0.032947	–	0.000	0.0207	0.0452

Table 3 shows that over the last 5 years, stillbirths among reproductive-age women increased. It is clear that the increase explained by the coefficient effect of selected independent variables is more important (0.026742), indicating that there is an 81.17% total change in stillbirths from 2012 to 2017 than the part of the endowment that showed a change in stillbirth prevalence of 18.83%. The results of overall decomposition revealed that the increase in stillbirths was due to behavioral changes rather than the composition of independent variables.

Instead of a rise explained by endowments, the overall increment was significantly increased by the coefficient effect, and each factor's contribution varied greatly according to categories (Table 4). The factors that changed the effect of these indicators on stillbirths for the two time points were women's wealth status, maternal education, delivery method, and geography (Sindh and Balochistan), as well as community maternal education and community wealth index.

**Table 4:** Details of multivariate decomposition of factors differences in stillbirths, PDHS from 2012 to 2013 and 2017 to 2018.

**Characteristics**	**Endowments**		**Coefficients**	
	**Coefficient**	**Percentage**	**Coefficient**	**Percentage**
**Media exposure (ref**. = **No)**				
**Yes**	−0.000001 [−0.00066, 0.000064]	−0.003	−0.004901 [−0.01886, 0.00906]	−14.88
**Wealth index (ref**. = **poor)**				
Middle	0.0004319 [−0.00025, 0.00113]	1.31	−0.003381 [−0.00896, 0.002201]	−10.26
Rich	0.000626 [−0.00046, 0.00172]	1.90	−0.01158 [−0.02291, 0.00024]^*^	−35.14
**Maternal age (ref**. = **15–24)**				
25–29	−0.000989 [−0.00267, 0.00069]	−3.00	0.006895 [−0.00527, 0.01907]	20.93
30+	−0.001201 [−0.00314, 0.00074]	−3.65	0.01427 [−0.01027, 0.03880]	43.30
**Maternal educated (ref**. = **no education)**				
Primary	−0.000077 [−0.00034, 0.00018]	−0.23	0.005857 [0.00115, 0.01056]^*^	17.78
Secondary	−0.000581 [−0.00177, 0.00061]	−1.76	0.008480 [0.00318, 0.01378]	25.74
Higher	−0.000014 [−0.00070, 0.00067]	−0.04	0.00641 [0.002800, 0.01002]^*^	19.46
**Maternal work status (ref**. = **unemployed)**				
Employed	0.000432 [−0.00039, 0.00126]	1.31	−0.003493 [−0.00958, 0.00259]	−10.60
**Father education (ref**. = **un-educated)**				
Educated	0.000130 [−0.00024, 0.00050]	0.40	−0.002333 [−0.01504, 0.01037]	−7.08
**Birth order (ref**. = **1–3)**				
4–6	0.000023 [−0.00028, 0.00032]	0.07	0.003382 [−0.00478, 0.01155]	10.26
7+	0.0002033 [−0.00022, 0.00062]	0.62	0.003463 [−0.00135, 0.00828]	10.51
**Birth interval (ref**. = < **24 months)**				
24–36 Months	−0.000004 [−0.00018, 0.00017]	−0.01	0.002944 [−0.00481, 0.01017]	8.94
48+ Months	−0.000361 [−0.00098, 0.00026]	−1.09	0.003345 [−0.00348, 0.01017]	10.15
**Place of delivery (ref**. = **home)**				
Health Facility	−0.001086 [−0.00384, 0.00167]	−3.29	0.004031 [−0.00470, 0.01277]	12.24
**Mode of delivery (ref**. = **vaginal)**				
Cesarean Section	−0.000067 [−0.00086, 0.00073]	−0.20	−0.00298 [−0.00608, 0.00013]^**^	−9.03
**Place of residence (ref**. =**urban)**				
Rural	−0.000479 [−0.00137, 0.00042]	−1.45	0.016207 [−0.00397, 0.03639]	49.19
**Region (ref**. = **Punjab)**				
Sindh	−0.000102 [−0.00034, 0.00013]	−0.31	0.00464 [−0.00021, 0.00950]^**^	14.10
KPK	0.000138 [−0.00048, 0.00076]	0.42	0.000832 [−0.00287, 0.00950]	2.53
Balochistan	0.000111 [−0.00007, 0.00029]	0.34	−0.00144 [−0.00305, 0.00017]^**^	−4.37
ICT	0.000003 [−0.00004, 0.000048]	0.01	−0.000049 [0.00020, 0.00010]	−0.15
**Maternal education (ref**. = **low level)**				
High level	0.000973 [−0.00072, 0.00266]	2.95	−0.013292 [−0.02242, 0.00416]^*^	−40.34
**Wealth index (ref**. = **low level)**				
High	−0.001012 [−0.00278, 0.00076]	−3.07	0.032647 [0.01741, 0.04788]^*^	99.09
**Media exposure (ref**. = **low level)**				
High	−0.000460 [−0.00150, 0.00057]	−1.39	0.00887 [−0.00220, 0.01995]	26.94
Intercept	**–**	**–**	−0.05209 [−0.11011, 0.00592]^**^	−158.12

The component of the change in stillbirth attributed to wealth status revealed that if the 2012–2013 PDHS data were adjusted for community wealth status to the same degree as the 2017–2018 PDHS data, the 2017–2012 gap in stillbirths would be expected to decrease by 35.14% due to behavioral changes..

The proportion of educated mothers increased during the study, and this had a substantial impact on the decline in stillbirths. Approximately 17.78% of the difference was due to behavioral change among mothers who received their primary education. According to the coefficient estimate for highly educated mothers, which was 0.00641, the log rate of stillbirth would have increased by 19.46% between 2012 and 2017 if nothing else in the model had changed.

However, due to the effects of the explanatory variable, an increase in cesarean section deliveries resulted in a gradual drop (−0.002975) in stillbirth. The change in stillbirths for mothers who underwent cesarean sections was 9.03%. Cesarean deliveries negatively impacted stillbirths with a log rate of −0.000067, which was due to disparities in endowments.

A considerable increase in stillbirths was observed for women who lived in rural areas (49.19%) compared to those who did not (due to behavioral changes). However, endowment projections showed a slight decline in stillbirths in rural areas due to a shift in characteristics. Balochistan, however, showed a significantly smaller change in stillbirth prevalence over time, by 4.37%, due to the difference in coefficients, with a contribution from characteristics/composition of 0.34%. Between 2012 and 2017, women who lived in or gave birth in the Sindh region contributed 14.10%, a 0.004644% increase in the log rate of stillbirth.

Communities with high levels of maternal education helped reduce the stillbirth rate by −0.013292 between 2012 and 2017 due to behavioral adjustments. According to the coefficient estimate, mothers who lived in neighborhoods with high levels of education had a 40.34% lower lifetime risk of stillbirth. The endowment component, however, indicated a 2.95% reduction between the 2 survey years.

Similarly, a rise in the proportion of wealthy communities from 2012 to 2017 considerably influenced the shift in stillbirth rates, which increased by 99.09%. The estimation of a population with a high level of wealth showed that the coefficient difference increased the likelihood of stillbirths. While the endowment estimate demonstrated the impact of differences in characteristics, it also showed that a reduction in the proportion of women living in wealthy communities contributed to a 3.07% decrease in stillbirths.

The results of compositional differences showed that older mothers had small, unfavorable changes (−3.65%), while the effects showed that older women had the highest rise in stillbirths.

In Table 4, the endowment section illustrates that over time, women with higher levels of education had a lower chance of stillbirth. The number of stillbirths changed by 0.23% for mothers with primary education, 1.76% for mothers with secondary education, and 0.04% for mothers with higher levels of education.

## Discussion

The discussion was carried out by dividing the study's outcomes into four thematic areas. It was observed that a few issues affecting women, supply-side gaps, awareness gaps, and government policy gaps contributed to the rise in the stillbirth rate over time.

### Issues affecting women

Women's socioeconomic backgrounds may play a role in the likelihood of stillbirths. Many Pakistani women gave birth even at a young age. Older women are more likely to have stillbirths as compared to those who give birth vaginally. Education and labor force participation rates among women in Pakistan remain low. In both surveys, Pakistani women experienced short birth intervals, which might be due to their limited participation in economic activity. High parity and short birth intervals also contribute to the increased percentage of stillbirths. Recent studies have also revealed that the percentage of overweight mothers is higher than in previous surveys. Overweight mothers in Pakistan are more susceptible to the risk of stillbirths.

Higher education for mothers is a protective factor. Stillbirths decreased among highly educated women over the entire study period compared to uneducated women due to the change in characteristics, but a surprising result from the behavioral changes showed that highly educated women contributed more to the change in stillbirth rates from 2012 to 2017. This surprising result of a higher uptake of stillbirth among highly educated women might be because uneducated mothers may underreport stillbirths due to social stigmas and taboos (16). However, other studies found that the risk of stillbirth was less likely to occur among educated mothers (37–39).

### Supply side gaps

The supply-side gap in the provision of accessible and affordable healthcare services is prevalent for many reasons. The majority of women had vaginal deliveries. Despite the availability of prenatal care, some women failed to attend any antenatal appointments. These women were found to have a higher risk of stillbirths. Poverty is also responsible, as healthcare facilities are not affordable for poor women.

Women with high socioeconomic status contributed 35% to the reduction of stillbirths. Individual wealth status mostly plays a negative role in stillbirths (40, 41). This result indicated that women with higher wealth status exhibited a larger uptake than those with poor health facilities.

An increase in cesarean deliveries had a significant effect on the reduction in stillbirths. This finding is consistent with a prior study conducted in Ethiopia (24). This is because the cesarean section is performed in an advanced health facility that minimizes pregnancy complications (42). The decline that was observed in decomposition analysis is particularly low. This indicated that there was still some risk for Pakistani mothers who chose cesarean sections. This might be because maternal health services in developing countries, including Pakistan, are not easily available, especially in rural areas. More than 60% of Pakistan's population lives in rural areas, where hospitals lack adequate health facilities and equipment to perform safe deliveries in the event of complications. Improved quality of childbirth care in public hospitals can solve many problems and will greatly help build women's trust in a health facility (43).

A rise in the proportion of wealthy communities showed that these communities contributed more to stillbirths. Surprisingly, a community with a high level of wealth found that behavioral changes raised the likelihood of stillbirth. Empirical research in Bangladesh and other countries has shown that socioeconomic circumstances play a significant role in the pursuit of community health care (44). While this is a startling and unexpected conclusion, the results are not in line with those of earlier research (4). The results of the community wealth status were affected by cluster variance or any other unmeasured factor. This may be because groups with higher wealth status tend to have lower education levels and reside in rural areas with inferior healthcare infrastructure. Pakistan, a developing country, has socioeconomic constraints that cause some rural areas to have health issues (45). Poor socioeconomic standing is frequently a barrier to receiving quality medical care (46, 47). Transportation costs, pricey medications, and medical fees can all be obstacles.

### Awareness gaps

The media is one of the primary sources of awareness, including health-related awareness. Studies have shown that media exposure can be beneficial for Pakistani mothers. However, the percentage of women who had no media exposure highlights a gap in awareness.

Women who resided in highly educated neighborhoods contributed to the drop in stillbirths substantially. The effect of this community-level education is different from the effect of education at the individual level. This outcome at the community level can be attributed to the fact that communities with high levels of education can enhance a person's health-seeking behavior. In these communities, healthcare facilities are also available and accessible. For example, women are better able to take care of their own health and that of their unborn children. They are more aware of pregnancy complications and use maternal health care promptly due to greater awareness in the communities and a higher level of education. This outcome is consistent with other investigations (37, 38). The awareness gap also exists, as the majority of Pakistani women are not following optimal birth spacing due to a lack of awareness of the risks of short birth intervals between two successive births.

### Government policy gaps

Stillbirths have increased in Pakistan over the last 2 decades. To elucidate the elements that contribute to the shift in stillbirths, it is crucial for public health to identify the reasons for the change. The increase in the stillbirth rate over the study period, which was 81.2%, can be linked more to changes in the overall effect of factors or indicators than to changes in endowments or compositional change of prevalence between the two points of time, according to the results of the multivariate decomposition technique.

Stillbirths are observed more frequently for women residing in Punjab, Sindh, and rural areas. It indicates that there is a gap in government policy to supply healthcare facilities in each region based on equity.

## Conclusion and recommendations

Stillbirths increased in Pakistan from 2012 to 2017. Stillbirths were observed more frequently for women residing in Punjab, Sindh, and rural areas. Older, overweight, and poor women are more likely to have stillbirths than those who give birth vaginally. The risk of stillbirths was more pronounced for women who had had antenatal care. High parity and short birth intervals also accelerated the rates of stillbirths.

The higher economic status of women, cesarean sections, and communities with higher levels of women's education significantly decreased the number of stillbirths in Pakistan. An effective strategy to curb stillbirths is to offer women accessible and affordable healthcare services. Awareness campaigns for the health education of pregnant women should focus on raising awareness to support better pregnancy outcomes for poor women living in communities with low levels of women's education. Offering free diagnostics for the early detection of birth complications in low-resource settings and referring these cases to knowledgeable gynecologists for safe delivery can lower the risk of stillbirth.

## Data availability statement

Publicly available datasets were analyzed in this study. This data can be found here: PDHS data of 2012213 and 2017-2018.

## Ethics statement

The study doesn't involve the collection of information from subjects. Consent to participate is not applicable. Since the study is a secondary data analysis based on DHS data.

## Author contributions

The conception of the work, design of the work, acquisition of data, analysis, interpretation of data, data curation, drafting the article, revising it critically for intellectual content, validation, and final approval of the version to be published was done AS, AK, MI and GAT. Helped out the paper in the revision stage, critically review the paper, and fix all typos and grammatical mistakes was done by TA. All authors read and approved the final manuscript.
